# Band Gaps Characteristics Analysis of Periodic Oscillator Coupled Damping Beam

**DOI:** 10.3390/ma13245748

**Published:** 2020-12-16

**Authors:** Li Tang, Xiongliang Yao, Guoxun Wu, Dong Tang

**Affiliations:** 1 College of Shipbuilding Engineering, Harbin Engineering University, Harbin 150001, China; tanglitly@hrbeu.edu.cn (L.T.); xiongliangyao@hrbeu.edu.cn (X.Y.); 2 College of Harbour Coastal and Offshore Engineering, Hohai University, Nanjing 210098, China; tangdong@hhu.edu.cn

**Keywords:** periodic oscillator coupled beam, flexural wave vibration band gap, band gap vibration attenuation, method of reverberation-ray matrix

## Abstract

The vibration of the periodic oscillator coupled damping beam model is reduced through the band gaps designing method, which can be applied in equivalent engineering structures. In this paper, the flexural wave dispersion relations of the infinite long periodic oscillator coupled damping beam were calculated using the reverberation-ray matrix method combined with the Bloch theorem. The flexural wave vibration frequency response function of the finite long periodic oscillator coupled damping beam was carried out using the finite element method. The flexural wave vibration band gaps occur in the infinite long periodic oscillator coupled damping beam model in both the analytical and numerical results. In these band gaps, flexural waves’ propagation is prohibited, and flexural vibration is significantly suppressed. Furthermore, the effects of structure and material parameters on the flexural wave vibration band gaps characteristics are studied. Thus, the structural vibration reduction design can be realized by adjusting the related parameters of the periodic coupled damping beam structures and the equivalent 2D periodic stiffened plate structures.

## 1. Introduction

As a common basic structure in engineering, various forms of beam structures are widely used in civil engineering, mechanical power engineering, aerospace, naval architecture, and ocean engineering. The beam structures’ design method has been one of the main research objects in the field of vibration and noise control for many years [1,2,3]. Many scholars have done a lot of work in this field, trying to reduce vibration and noise through different methods [4,5,6,7,8]. Among these methods, phononic crystals have been introduced into the structural vibration reduction design due to the advantage of band gap characteristics [9,10,11,12].

Phononic crystals were proposed by Yablonovitch [13] and John [14]. The phononic crystals are composed of two or more units with different materials or configurations that are periodically arranged. Elastic wave propagation of phononic crystals is prohibited in specific frequency ranges called band gaps or forbidden bands [15]. Existing research of phononic crystals has revealed two band gap formation theories: Bragg scattering band gap and local resonance band gap. The periodicity of the structure mainly determines the Bragg scattering band gap. The local resonance of the microstructure mainly determines the local resonance band gap in the phononic crystals cell. Local resonance phononic crystals were first explicitly proposed by Liu et al. in 2000 [16]. They fabricated a three-dimensional three-component local resonant phononic crystals by embedding a periodically arranged microstructure unit with low-frequency resonance characteristics in a poly material. Both the theoretical and experimental studies by Liu et al. have confirmed that these phononic crystals can produce a low-frequency local resonance band gap that is much lower than the traditional Bragg gap frequency [17]. Due to the remarkable band gap characteristics of elastic wave propagation in phononic crystals, it has been widely studied by a large number of scholars and applied to engineering design as a vibration-damping element [18].

From the perspective of scale, the phononic crystals extend infinitely in the three-dimensional direction, which is an ideal mathematical model. However, for engineering applications, the phononic crystals degenerate into a finite-scale periodic structure in at least one direction. The studies of phononic crystals have developed from microscopic photonic crystals to the field of engineering and promoted its development in two fields [19]. The latest approaches for the phononic crystals can be widely used to study nanoparticles’ dynamics and microscale granular crystals [20,21]. At present, the main research objects of finite-scale periodic structures in the engineering field include periodic composite beams [22,23], periodic dynamic absorber coupled beams [24,25], periodic plates [26,27], periodic oscillator coupled plates [28,29], and periodic composite cylindrical shells [30].

Yu et al. verified the Bragg gap and local resonance band gap of beam periodic structures from the aspects of theory, simulation, and experiment [31]. Wang has studied simplified models of two-dimensional and three-dimensional phononic crystals [32], and the results show that the base and local oscillators can be simplified into one-dimensional periodic oscillator coupled beam, which is a periodic spring-mass oscillator coupled homogeneous straight damping beam combined system. Cho et al. [33] investigated free vibration numerical analysis of one-dimensional stiffened plates with multiple lumped mass and stiffness attachments. Zhou et al. [34] investigated the flexural wave propagation characteristics in the one-dimensional periodically stiffened plate, and the results showed that one-dimensional periodically stiffened plate could yield complete band gaps. The periodic oscillator coupled beam and periodic stiffened plates are typical periodic structures with periodic band gap characteristics. As basic constitutive members of bridge structures, ships, and offshore structures, periodic one-dimensional stiffened plates and periodic bi-directionally orthogonal stiffened plates are also widely used in civil engineering, naval architecture, and ocean engineering. To some extent, the unidirectional low-frequency vibration characteristics of periodic stiffened plates commonly used in engineering are like those of the periodic oscillator coupled beams. Thus, studying the band gap characteristics of the periodic oscillator coupled beams and introducing them into the design of the bending vibration beam structures are of great significance to the research on the vibration control of beam structures and periodic stiffened plates in engineering.

The calculation and analysis of band gaps are the basis and important issues of periodic structure research. Existing calculation methods mainly include the plane wave expansion method [35], finite-difference time-domain method [36], multiple-scattering method [37], and lumped mass method [38]. Due to the complexity of periodic bi-directionally orthogonal stiffened plates and common engineering structures, there are no effective analytical methods to study these structures’ dynamics. The wave finite element method is a commonly used approach to calculating the band gaps and transmission characteristics of complex periodic structures [39,40]. The lumped mass method has the advantages of high accuracy and good convergence, which is especially suitable for band gap calculation of one-dimensional periodic structures. One-dimensional periodic structures can be equivalent to periodic oscillators by using the lumped mass method. Oscillators’ mass and spring stiffness are determined by the equivalent elastic modulus, length, and cross-sectional dimensions of the one-dimensional periodic structures. Thus, the periodic stiffeners along the breadth direction of the periodic bi-directionally orthogonal stiffened plate can be equivalent to periodic oscillators, the stiffeners along the length direction and the plate can be equivalent to homogeneous straight beam, and then the periodic bi-directionally orthogonal stiffened plate can be equivalent to a periodic oscillator coupled beam. Existing studies by Richards et al. have shown that the material and geometric parameters of the periodic structure’s elements can be appropriately selected to adjust the band gap range of the periodic structure [41]. Smart materials such as piezoelectric crystals [42], shape memory alloys [43], and functionally graded materials [44] can also be used to change the elastic modulus actively and then adjust the frequency ranges of band gaps.

Obviously, the current research on band gaps mainly focuses on the positive problems but designing the periodic structure to modulate the band gaps and vibration attenuation properties in specific frequency ranges is rarely involved. Numerical and experimental methods are commonly used analysis tools to study the band gaps and transmission characteristics of periodic bi-directionally orthogonal stiffened plates; it is hard to theoretically investigate the effects of parameters on band gaps and guide the vibration reduction design. Therefore, it is of great theoretical significance and engineering application value to study periodic structure parameters’ influence on band gaps. As a preliminary discussion of periodic structure band gaps and vibration attenuation properties design, this paper derived the calculation method of the flexural wave vibration band gaps of infinite long periodic oscillator coupled damping beam by the method of the reverberation-ray matrix in combination with Bloch theorem. The reverberation-ray matrix method is a semi-analytical method and the advantages of the present method lie in its simplicity, clarity, and accuracy [45]. Furthermore, we investigate the influences of the various structural parameters of the periodic oscillator coupled damping beam on the band gaps characteristics, including the effects of the damping. This research work provides an important reference for the vibration reduction design of beam structures and the equivalent 2D periodic stiffened plate structures, which are widely used in aerospace, naval architecture, and ocean engineering.

## 2. Physics Model and Calculation

**Figure 1:**
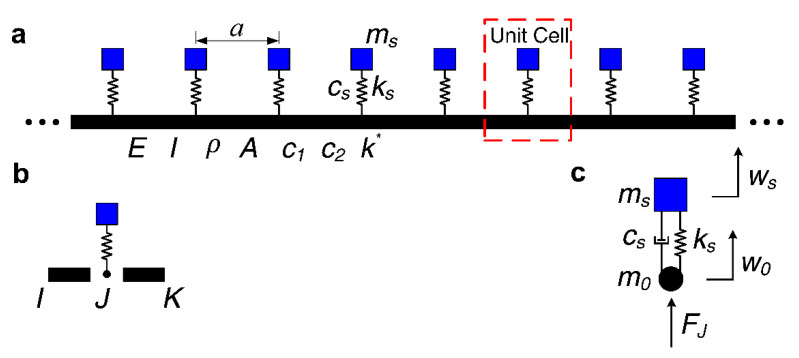
The physics model: (**a**) Schematic diagram of infinite long periodic oscillator coupled damping beam; (**b**) schematic diagram of the unit cell; (**c**) coupling system of spring-mass oscillator and the beam node in a unit cell.

The physical model of infinite long periodic oscillator coupled damping beam considered in this paper is constituted of periodic spring-mass oscillators coupled an infinitely long homogeneous straight damping beam as illustrated in Figure 1a. The area surrounded by a red dashed line in Figure 1a is the unit cell of the proposed infinite long periodic oscillator coupled damping beam and the schematic diagram is shown in Figure 1b, and the coupling system of spring-mass oscillator and beam node in a unit cell is plotted in Figure 1c. The infinite long periodic oscillator coupled damping beam can be obtained by periodically repeating and combining the unit cell along the length direction. The lattice constant of the proposed infinite long periodic oscillator coupled damping beam is defined as *a*. *m_s_* is the mass of the spring-mass oscillator, *k_s_* and *c_s_* respectively stand for the spring stiffness and the damping of the spring-mass oscillator; *E* is Young’s modulus, *I* is the section moment of inertia, *ρ* is the mass density, and *A* denotes the section area of the homogeneous straight damping beam; k* stands for the support stiffness of the elastic foundation, *c*_1_ and *c*_2_ are the translational damping and rotational damping of the homogeneous straight damping beam, respectively. To investigate the flexural wave propagation and vibration band gaps in the proposed infinite long periodic oscillator coupled damping beam, the reverberation-ray matrix combined with Bloch theorem was applied to calculate the flexural wave dispersion relations.

As per the physics model plotted in Figure 1b, the unit cell of infinite long periodic oscillator coupled damping beam is constituted of beam *IJ*, beam *JK* is connected the spring-mass oscillator at the node *J.* Thus, the major structure in a unit cell is the damping beams, and the spring-mass oscillator can be considered as the boundary condition of the beams. Based on the displacement continuous and force equilibrium relationships of the unit cell structures at the connection point between the beams and the spring-mass oscillator, the local scattering relationship at the node *J* can be obtained. According to the periodic structure Bloch theorem of node *I* and node *K*, the local scattering relationships at the node *J* and node *K* would be obtained; thus, the unit cell’s overall scattering relationship would be obtained. The unit cell’s overall phase relationship can be obtained according to the local phase relationship in beam *IJ* and beam *JK*. Finally, the reverberation-ray matrix can be obtained by combining the scattering relationship, the phase relationship and the permutation relationship. Then, the unit cell’s flexural wave dispersion relations will be obtained, which is the flexural wave vibration band gaps of the proposed infinite long periodic oscillator coupled damping beam.

If the cross-section size is not small compared with the length of the beam, the effects of the moment of inertia and shear deformation need to be in consideration. According to the shear correction theory of the beam, the bending vibration governing equations of a homogeneous straight Timoshenko damping beam with elastic foundation support are given as follows [46]:

(1) $$EI\frac{\partial^{2}\varphi }{\partial x^{2}}+\kappa GA\left(\frac{\partial w}{\partial x}-\varphi \right)-c_{2}\frac{\partial \varphi }{\partial t}-\rho I\frac{\partial^{2}\varphi }{\partial t^{2}}=0$$

(2) $$\kappa GA\left(\frac{\partial^{2}w}{\partial x^{2}}-\frac{\partial \varphi }{\partial x}\right)-c_{1}\frac{\partial w}{\partial t}-\rho A\frac{\partial^{2}w}{\partial t^{2}}+k^{*}w=0$$

where *G* = *E*/[2(1 + *ν*)] is the shear elastic modulus of the beam material, *ν* represents the Poisson’s ratio, *κ* is the Timoshenko shear correction coefficient, *w* and *φ* respectively represent the vertical displacement and rotation angle of the beam, *t* denotes the time, and *x* represents the one-dimensional space coordinate variable in Cartesian coordinate system.

Using the simultaneous Equations Equations (1) and (2) and eliminating the rotation angle variable *φ*, the movement differential equation of the proposed beam expressed only by vertical displacement *w* can be obtained as

(3) $$\begin{array}{l} \frac{\partial^{4}w}{\partial x^{4}}+\frac{k^{*}}{\kappa GA}\frac{\partial^{2}w}{\partial x^{2}}-\left(\frac{c_{1}}{\kappa GA}+\frac{c_{2}}{EI}\right)\frac{\partial^{3}w}{\partial x^{2}\partial t}-\left(\frac{\rho }{\kappa G}+\frac{\rho }{E}\right)\frac{\partial^{4}w}{\partial x^{2}\partial t^{2}}+\left(\frac{c_{1}}{EI}-\frac{c_{2}k^{*}}{EI\kappa GA}\right)\frac{\partial w}{\partial t}\\ +\left(\frac{\rho A}{EI}+\frac{c_{1}c_{2}}{EI\kappa GA}-\frac{\rho k^{*}}{E\kappa GA}\right)\frac{\partial^{2}w}{\partial t^{2}}+\left(\frac{\rho c_{2}}{EI\kappa G}+\frac{\rho c_{1}}{E\kappa GA}\right)\frac{\partial^{3}w}{\partial t^{3}}+\frac{\rho^{2}}{E\kappa G}\frac{\partial^{4}w}{\partial t^{4}}-\frac{k^{*}}{EI}w=0 \end{array}$$

The waveform solution of the proposed beam’s vertical displacement can be expressed in the form of *w* = *W*_0_*e*^i(*kx*−*ωt*)^. Substituting it into Equation (3), and the characteristic equation corresponding to the waveform solution can be written as

(4) $$\begin{array}{l} k^{4}+\left[-\frac{K^{*}}{\kappa GA}-i\omega \left(\frac{c_{1}}{\kappa GA}+\frac{c_{2}}{EI}\right)-\omega^{2}\left(\frac{\rho }{\kappa G}+\frac{\rho }{E}\right)\right]k^{2}-i\omega \left(\frac{c_{1}}{EI}-\frac{c_{2}K^{*}}{EI\kappa GA}\right)\\ -\omega^{2}\left(\frac{\rho A}{EI}+\frac{c_{1}c_{2}}{EI\kappa GA}-\frac{\rho K^{*}}{E\kappa GA}\right)+i\omega^{3}\left(\frac{\rho c_{2}}{EI\kappa G}+\frac{\rho c_{1}}{E\kappa GA}\right)+\frac{\rho^{2}}{E\kappa G}\omega^{4}-\frac{K^{*}}{EI}=0 \end{array}$$

where *k* is the complex wavenumber and *ω* is the circular frequency.

Equation (4) has four roots in the complex field, which are given respectively by

(5) $$k_{1,3}=\pm {\sqrt{-\alpha /2+\sqrt{{\left(\alpha /2\right)}^{2}-\beta }}}_{}\;k_{2,4}=\pm \sqrt{-\alpha /2-\sqrt{{\left(\alpha /2\right)}^{2}-\beta }}$$

where $\alpha =-\frac{k^{*}}{\kappa GA}-i\omega \left(\frac{c_{1}}{\kappa GA}+\frac{c_{2}}{EI}\right)-\omega^{2}\left(\frac{\rho }{\kappa G}+\frac{\rho }{E}\right)$,

$$\beta =-i\omega \left(\frac{c_{1}}{EI}-\frac{c_{2}k^{*}}{EI\kappa GA}\right)-\omega^{2}\left(\frac{\rho A}{EI}+\frac{c_{1}c_{2}}{EI\kappa GA}-\frac{\rho k^{*}}{E\kappa GA}\right)+i\omega^{3}\left(\frac{\rho c_{2}}{EI\kappa G}+\frac{\rho c_{1}}{E\kappa GA}\right)+\frac{\rho^{2}}{E\kappa G}\omega^{4}-\frac{k^{*}}{EI}$$

Therefore, if the simple harmonic time factor *e*^−i*ωt*^ is omitted, the solution in the complex field of Equation (3) can be expressed as follows:

(6) $$w=a_{1}e^{ik_{1}x}+d_{1}e^{-ik_{1}x}+a_{2}e^{ik_{2}x}+d_{2}e^{-ik_{2}x}$$

Thus, the angle of the beam can be expressed as

(7) $$\varphi =g_{1}a_{1}e^{ik_{1}x}-g_{1}d_{1}e^{-ik_{1}x}+g_{2}a_{2}e^{ik_{2}x}-g_{2}d_{2}e^{-ik_{2}x}$$

where the expression of *g_j_* (*j* = 1, 2) is

(8) $$g_{j}=\frac{ik_{j}\kappa GA}{EIk_{j}^{2}+\kappa GA-ic_{2}\omega -\rho I\omega^{2}}$$

According to the relationship between shear force and bending moment with displacement and rotation angle in Timoshenko beam, which is given as

(9) $$M=EI{\frac{\partial^{2}w}{\partial x^{2}}}_{}\;V=\kappa GA\left(\varphi -\frac{\partial w}{\partial x}\right)$$

the expressions of shear and bending moment can be obtained as follows:

(10) $$M=-EI\left(k_{1}^{2}a_{1}e^{ik_{1}x}+k_{1}^{2}d_{1}e^{-ik_{1}x}+k_{2}^{2}a_{2}e^{ik_{2}x}+k_{2}^{2}d_{2}e^{-ik_{2}x}\right)$$

(11) $$V=\kappa GA\left[\left(g_{1}-ik_{1}\right)a_{1}e^{ik_{1}x}-\left(g_{1}-ik_{1}\right)d_{1}e^{-ik_{1}x}+\left(g_{2}-ik_{2}\right)a_{2}e^{ik_{2}x}-\left(g_{2}-ik_{2}\right)d_{2}e^{-ik_{2}x}\right]$$

The Equations (6), (7), (10) and (11) can be rewritten in matrix form respectively as

(12) $$W_{d}=A_{d}P_{h}\left(-x\right)a+D_{d}P_{h}\left(x\right)d$$

(13) $$W_{f}=A_{f}P_{h}\left(-x\right)a+D_{f}P_{h}\left(x\right)d$$

where ***W****_d_* = {*w*, *φ*}*^T^* and ***W****_f_* = {*V*, *M*}*^T^* respectively represent generalized displacement (including rotation angle) vector and generalized force (including moment) vector; ***a*** and ***d*** are the arrival wave amplitude vector and leaving wave amplitude vector, respectively; ***P****_h_* is the phase matrix, ***A****_d_* and ***D****_d_* respectively stand for the arriving wave coefficient matrix and the leaving wave coefficient matrix corresponding to the displacement vector, and ***A****_f_* and ***D****_f_* respectively denotes the arriving wave coefficient matrix and the leaving wave coefficient matrix corresponding to the force vector. The specific expressions of them are given as follows:

(14) $$W_{d}={\left\{\begin{array}{cc} w & \varphi \end{array}\right\}}^{T}\;W_{f}={\left\{\begin{array}{cc} V & M \end{array}\right\}}^{T}$$

(15) $$a={\left\{\begin{array}{cc} a_{1} & a_{2} \end{array}\right\}}^{T}\;d={\left\{\begin{array}{cc} d_{1} & d_{2} \end{array}\right\}}^{T}$$

(16) $$P_{h}\left(x\right)=\left[\begin{array}{cc} e^{-ik_{1}x} & 0\\ 0 & e^{-ik_{2}x} \end{array}\right]$$

(17) $$A_{d}={\left[\begin{array}{cc} 1 & 1\\ g_{1} & g_{2} \end{array}\right]}_{}\;D_{d}=\left[\begin{array}{cc} 1 & 1\\ -g_{1} & -g_{2} \end{array}\right]$$

(18) $$A_{f}=\left[\begin{array}{cc} \kappa GA & 0\\ 0 & -EI \end{array}\right]{\left[\begin{array}{cc} g_{1}-ik_{1} & g_{2}-ik_{2}\\ k_{1}^{2} & k_{2}^{2} \end{array}\right]}_{}\;D_{f}=\left[\begin{array}{cc} \kappa GA & 0\\ 0 & -EI \end{array}\right]\left[\begin{array}{cc} ik_{1}-g_{1} & ik_{2}-g_{2}\\ k_{1}^{2} & k_{2}^{2} \end{array}\right]$$

As the coupling system of spring-mass oscillator and the beam node in the infinite long periodic oscillator coupled damping beam unit cell plotted in Figure 1c, the relationships of the displacement continuity and the force equilibrium at the internal node *J* of the unit cell can be expressed as follows:

(19) $$W_{d}^{JI}=T_{d}^{J}W_{d}^{JK}$$

(20) $$W_{f}^{JI}=T_{f}^{J}W_{f}^{JK}+F^{J}$$

where $T_{d}^{J}=diag\left\{\begin{array}{cc} -1 & 1 \end{array}\right\}$ and $T_{f}^{J}=-T_{d}^{J}$ respectively represent the generalized displacement transformation matrix and generalized force transformation matrix at node *j*, ***F****^J^* stand for the reaction force vector of spring-mass oscillator acting on the beams.

As the coupling system of spring-mass oscillator and beam node plotted in Figure 1c, based on the displacement continuous and force equilibrium relationships of the unit cell structures at the connection point between the beams and the spring-mass oscillator, the coupling vibration equation of the spring-mass oscillator and the beam at the connection node in a proposed unit cell can be expressed by

(21) $$\left\{ \begin{array}{l} m_{s}{\ddot{w}}_{s}+k_{s}(w_{s}-w_{0})+c_{s}\left({\dot{w}}_{s}-{\dot{w}}_{0}\right)=0\\ m_{0}{\ddot{w}}_{0}+k_{s}(w_{0}-w_{s})+c_{s}\left({\dot{w}}_{0}-{\dot{w}}_{s}\right)=F_{J} \end{array}\right.$$

Then, the reaction force vector of the spring-mass oscillator acting on the beam can be obtained as

(22) $$F^{J}=K^{J}W_{d}^{JK}$$

where ***K****^J^* = diag{*k_w_ k_φ_*} denotes the dynamic stiffness matrix of the spring-mass oscillator acting on the beam at the connection node, the *k_w_* = −*m_s_ω*^2^ (*k_s_ + ic*_s_*ω*)/(*k_s_* − *m_s_ω*^2^ *+ ic*_s_*ω*) − *m*_0_*ω*^2^ and *k_φ_* = 0 respectively represent the moving stiffness coefficient and rotation dynamic stiffness coefficient of the spring-mass oscillator acting on the beam at the connection node, *k_s_* and *m_s_* are the spring stiffness coefficient and mass of the oscillator, respectively; *m*_0_ is the mass of the beam node at the node *J*, and generally *m*_0_ = 0; *w_s_* and *w*_0_ = *w^JK^* = −*w^JI^* respectively stand for the vertical displacement of the oscillator and the beam node displacement at the node *J*.

Substituting the expressions of ***W****_d_* and ***W****_f_* into Equations (19), (20) and (22), the scattering relationship at node *J* can be obtained as follows:

(23) $$A^{J}a^{J}+D^{J}d^{J}=0$$

where ***a****^J^* = {(***a****^JI^*)*^T^* (***a****^JK^*)*^T^*}*^T^* and ***d****^J^* = {(***d****^JI^*)*^T^* (***d****^JK^*)*^T^*}*^T^* respectively represent the arriving wave amplitude vector and the leaving wave amplitude vector at node *J*, ***A****^J^* and ***D****^J^* are the corresponding coefficient matrixes, respectively, and the expressions of them are given as

(24) $$A^{J}=\left[\begin{array}{cc} A_{d}^{JI} & -T_{d}^{J}A_{d}^{JK}\\ A_{f}^{JI} & -(T_{f}^{J}A_{f}^{JK}+K^{J}A_{d}^{JK}) \end{array}\right]\;D^{J}=\left[\begin{array}{cc} D_{d}^{JI} & -T_{d}^{J}D_{d}^{JK}\\ D_{f}^{JI} & -(T_{f}^{J}D_{f}^{JK}+K^{J}D_{d}^{JK}) \end{array}\right]$$

According to the periodic structure Bloch theorem, the displacement vector and internal force vector at both ends of the unit cell of the proposed infinite long periodic oscillator coupled damping beam satisfy the periodic condition, namely,

(25) $$e^{iqa}W_{d}^{IJ}=T_{d}^{J}W_{d}^{KJ}$$

(26) $$e^{iqa}W_{f}^{IJ}=T_{f}^{J}W_{f}^{KJ}$$

Similarly, substituting the expressions of ***W****_d_* and ***W****_f_* into Equations (25) and (26), we can proceed as follows:

(27) $$A^{*J}a^{*J}+D^{*J}d^{*J}=0$$

where a*^J^ = {(***a****^IJ^*)*^T^* (***a****^KJ^*)*^T^*}*^T^* and d*^J^ = {(***d****^IJ^*)*^T^* (***d****^KJ^*)*^T^*}*^T^* respectively represent the arriving wave amplitude vector and the leaving wave amplitude vector at node *J*, A*^J^ and D*^J^ are the corresponding coefficient matrixes, respectively, and the expressions of them are given as

(28) $$A^{*J}=\left[\begin{array}{cc} e^{iqa}A_{d}^{IJ} & -T_{d}^{J}A_{d}^{KJ}\\ e^{iqa}A_{f}^{IJ} & -T_{f}^{J}A_{f}^{KJ} \end{array}\right]\;D^{*J}=\left[\begin{array}{cc} e^{iqa}D_{d}^{IJ} & -T_{d}^{J}D_{d}^{KJ}\\ e^{iqa}D_{f}^{IJ} & -T_{f}^{J}D_{f}^{KJ} \end{array}\right]$$

Combining Equations (23) and (27), the overall scattering relationship of the infinite long periodic oscillator coupled damping beam unit cell can be obtained as follows:

(29) Aa+Dd=0

where ***a*** = {(***a****^IJ^*)*^T^* (***a****^JI^*)*^T^* (***a****^JK^*)*^T^* (***a****^KJ^*)*^T^*}*^T^* and ***d*** = {(***d****^IJ^*)*^T^* (***d****^JI^*)*^T^* (***d****^JK^*)*^T^* (***d****^KJ^*)*^T^*}*^T^* respectively represent the general arriving wave amplitude vector and the general leaving wave amplitude vector of the unit cell, ***A*** and ***D*** denote the corresponding coefficient matrixes, and the expressions of them are given as follows:

(30) $$\begin{array}{l} A=\left[\begin{array}{cccc} e^{iqa}A_{d}^{IJ} & 0 & 0 & -T_{d}^{J}A_{d}^{KJ}\\ 0 & A_{d}^{JI} & -T_{d}^{J}A_{d}^{JK} & 0\\ 0 & A_{f}^{JI} & -(T_{f}^{J}A_{f}^{JK}+K^{J}A_{d}^{JK}) & 0\\ e^{iqa}A_{f}^{IJ} & 0 & 0 & -T_{f}^{J}A_{f}^{KJ} \end{array}\right]\\ D=\left[\begin{array}{cccc} e^{iqa}D_{d}^{IJ} & 0 & 0 & -T_{d}^{J}D_{d}^{KJ}\\ 0 & D_{d}^{JI} & -T_{d}^{J}D_{d}^{JK} & 0\\ 0 & D_{f}^{JI} & -(T_{f}^{J}D_{f}^{JK}+K^{J}D_{d}^{JK}) & 0\\ e^{iqa}D_{f}^{IJ} & 0 & 0 & -T_{f}^{J}D_{f}^{KJ} \end{array}\right] \end{array}$$

The same series of flexural wave in a section of beam is both the leaving wave (or arriving wave) of the left end node and the arriving wave (or leaving wave) of the right end node. The amplitudes of them are the same, while the phases are different.

The phase relationships in the beam of any section (e.g., section *J*) can be shown as follows:

(31) $$a^{JK}=P^{JK}d^{KJ}$$

(32) $$a^{KJ}=P^{JK}d^{JK}$$

where ***P****^JK^* = −***P****_h_*(*L^JK^*) is the phase matrix of section *J* of the beam.

According to the phase relationships of all the beam sections, the overall phase relationship can be obtained as follows:

(33) $$a=Pd^{*}$$

where d* = {(***d****^JI^*)*^T^* (***d****^IJ^*)*^T^* (***d****^KJ^*)*^T^* (***d****^JK^*)*^T^*}*^T^* is the rearranged overall leaving wave amplitude vector, and ***P*** = diag{***P****^IJ^* ***P****^IJ^* ***P****^JK^* ***P****^JK^*} is the overall phase matrix.

Comparing the overall leaving wave amplitude vectors d* and ***d*** of the infinite long periodic oscillator coupled damping beam unit cell, the two vectors have the same elements, while the elements’ arrangements order is different. Thus, we obtain the relationship between the overall leaving wave amplitude vectors d* and ***d*** as

(34) $$d^{*}=Ud$$

(35) $$U=\left[\begin{array}{cccc} 0 & I_{2} & 0 & 0\\ I_{2} & 0 & 0 & 0\\ 0 & 0 & 0 & I_{2}\\ 0 & 0 & I_{2} & 0 \end{array}\right]$$

where ***U*** represents the permutation matrix between the overall leaving wave amplitude vectors d* and ***d***, ***I***_2_ denotes the two-order unit matrix.

Substituting Equations (33) and (34) into Equation (29), the overall system equation of the infinite long periodic oscillator coupled damping beam unit cell can be obtained as follows:

(36) (APU+D)d=0

where ***R*** = ***APU*** + ***D*** represents the reverberation-ray matrix of the infinite long periodic oscillator coupled damping beam unit cell.

According to the necessary conditions for the existence of a non-zero solution to the overall leaving wave amplitude vector ***d***, namely, the determinant of its coefficient matrix is zero; thus, the flexural wave dispersion relations equation of the proposed infinite long periodic oscillator coupled damping beam unit cell can be obtained as follows:

(37) det(APU+D)=0

Therefore, the relationship between the flexural wave number *q* and frequency *f* in the physics model’s unit cell can be solved by Equation (37), which is the flexural wave dispersion relations and vibration band gaps of the proposed infinite long periodic oscillator coupled damping beam.

## 3. Results and Discussion

### 3.1. The Bad Gaps of Infinite Long Periodic Oscillator Coupled Damping Beam

This section discusses the flexural wave vibration band gaps characteristics of the infinite long periodic oscillator coupled damping beam. In order to facilitate the study of the band gaps characteristics and analyze the influences of parameters on the band gaps characteristics, as the calculation example, the material parameters of the homogeneous straight damping beam are considered as *E* = 2.1 × 10^11^ Pa, *ρ* = 7850 kg/m^3^ and *ν* = 0.28, respectively. The elastic foundation parameters have great effects in civil engineering and mechanical power engineering, while it can be ignored in aerospace, naval architecture, and ocean engineering. Thus, we set the support stiffness of the elastic foundation k* = 0 N/m. Other geometrical parameters of the homogeneous straight damping beam and periodic spring-mass oscillator are shown in Table 1.

**Figure 2:**
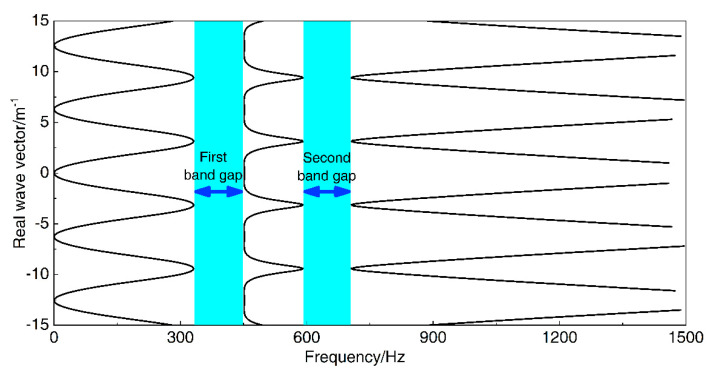
The flexural wave vibration band gaps structure of the proposed infinite long periodic oscillator coupled damping beam.

According to the bandgap calculation method described in the previous section, Figure 2 shows the calculated band gaps structure of the proposed infinite long periodic oscillator coupled damping beam in the calculation example. There are two band gaps of 331.1–451.3 Hz and 591.2–705.7 Hz in the frequency range from 0 to 1500 Hz, and the bandwidths are 120.2 Hz and 114.5 Hz, respectively.

### 3.2. Numerical Calculation Validation Based on Finite Element Method

**Figure 3:**
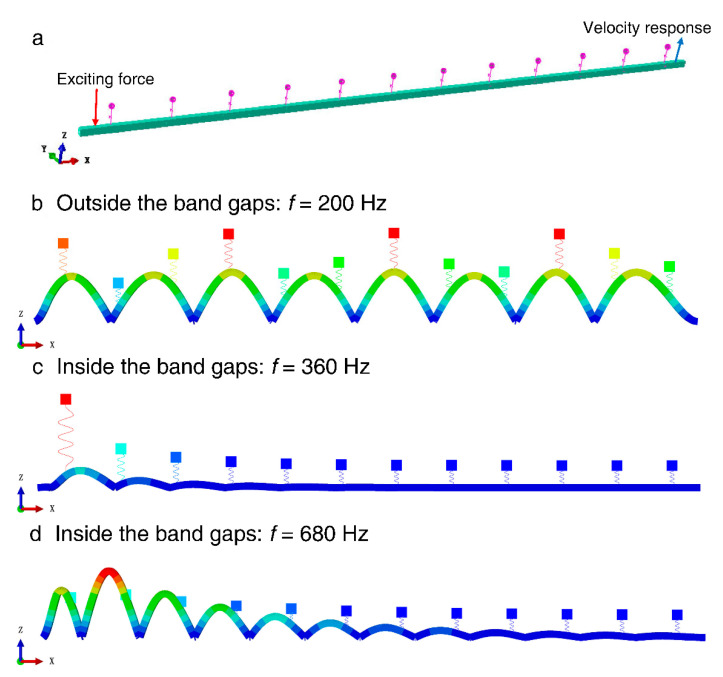
(**a**) Finite element model of the finite long periodic oscillator coupled damping beam with 12 unit cells; (**b**–**d**) the flexural wave vibration propagation characteristics at specific frequencies.

**Figure 4:**
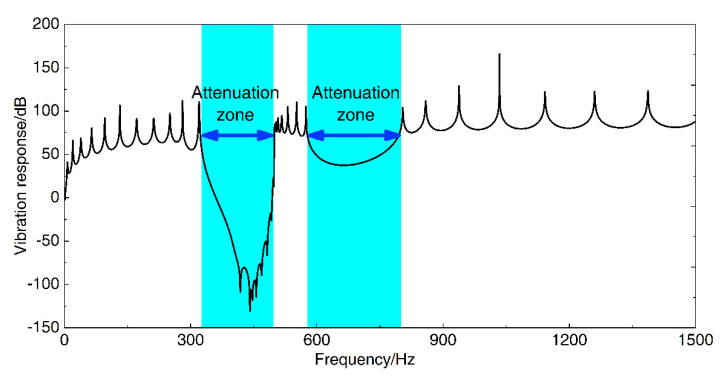
Band gaps and vibration attenuation properties represented by flexural wave vibration frequency response function.

In order to demonstrate the effectiveness of the calculation method of the flexural wave vibration band gaps derived in this paper and further prove the existence of the flexural wave vibration band gaps in the infinite long periodic oscillator coupled damping beam, the vibration transmission measurement experiment is the most precise and convincing method. However, the experimental method needs to spend a lot of manpower, material resources, and time to make it, and it is difficult to guarantee the accuracy of the experiment results. Fortunately, the finite element method is an effective and efficient calculation method of band gaps and vibration attenuation properties, the numerical calculation of finite array periodic structures by finite element method has been widely used to demonstrate the effectiveness of the band gaps calculation methods of periodic structures in the scholars’ research [47,48,49,50]. Thus, to validate the existence of the flexural wave vibration band gaps of the infinite long periodic oscillator coupled damping beam and study the vibration attenuation performance in the frequency ranges of band gaps, we investigated the band gaps and vibration attenuation characteristics by using the finite element method. Before the numerical calculation, a finite array finite element model composed of 12 unit cells of the proposed infinite long periodic oscillator coupled damping beam was established as shown in Figure 3a, the finite element model is constituted of 12 periodic spring-mass oscillators coupled with a straight beam with a rectangular section, which the vibration direction of the periodic spring-mass oscillators is vertical direction (Z-axis) the separation distance is 0.5 m denotes the lattice constant, and the length direction of the straight beam is along X-axis with 6 m dimensions. As a numerical calculation validation study, the material parameters and structural parameters of the finite element model were the same as the parameter settings in the study of the calculation example, which were considered as *E* = 2.1 × 10^11^ Pa, *ρ* = 7850 kg/m^3^, *ν* = 0.28, and listed in Table 1, respectively. During the numerical calculation, a unit exciting force with bandwidth from 0 to 1500 Hz was applied to perpendicularly stimulate the finite element model near the left end of the beam, and the vertical vibration velocity response was picked up near the right end of the beam to probe the transmitted flexural wave vibration signal. The schematic diagram of the exciting force and velocity response are shown in Figure 3a. Finally, the flexural wave vibration propagation characteristics at specific frequencies are shown in Figure 3b–d. Based on the spectral analysis and vibration signal processing, the flexural wave vibration band gaps and vibration attenuation properties are represented by the flexural wave vibration frequency response function, as shown in Figure 4.

There are two obvious vibration attenuations of 100 and 30 dB on average in the frequency bands of 322–498 and 583–779 Hz, respectively. The locations and bandwidths of the vibration attenuations in the flexural wave vibration frequency response function are in good agreement with the corresponding band gaps characteristics of the proposed infinite long periodic oscillator coupled damping beam, which is validated that the method for calculating and analyzing the bandgap characteristics described in this paper is effective. The flexural wave vibration propagation characteristics at 360 and 680 Hz shown in Figure 3c,d revealed the obvious vibration attenuations by the displacement of the right part of the beam, and Figure 3c,d also further demonstrate the effectiveness of the numerical calculation validation approach. In addition, it can be concluded that the flexural wave propagation within specific frequency ranges is forbidden, and obvious flexural wave vibration attenuations could appear in the infinite long periodic oscillator coupled damping beam, resulting in the significant reduction of structural vibration as shown in Figure 3c,d. Unlike the vibration attenuation caused by traditional material damping, the flexural wave vibration attenuations in periodic structures have relatively large significant vibration attenuations in specific frequency ranges, called band gaps. These vibration attenuations are caused by Bragg’s reflection and local resonance, which are mainly determined by the periodicity of the periodic structures and local resonance of the microstructure, respectively. However, the vibration attenuation caused by material damping is determined by the properties of material damping, which absorbs and consumes the energy of the flexural wave vibration, these attenuations would appear in a large frequency range, whereas the main effective area is medium/high frequency, and the vibration attenuation amplitudes are usually less than that in vibration attenuations band gaps.

### 3.3. Parametric Study

In order to analyze and study the effects of the parameters on the flexural wave vibration band gaps characteristics of the infinite long periodic oscillator coupled damping beam, the flexural wave dispersion relations of the proposed infinite long periodic oscillator coupled damping beam with different parameter values were performed by using the control variable method. During the parametric studies, the structural parameters listed in Table 1 were used to achieve the dimensionless parameter study.

#### 3.3.1. Effect of Spring Stiffness on the Band Gaps

**Figure 5:**
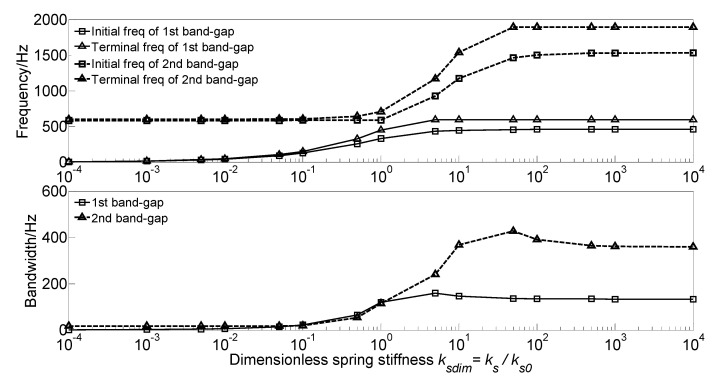
Band gaps maps as functions of dimensionless spring stiffness *k_sdim_*.

To investigate the effect of spring stiffness *k_s_* of the spring-mass oscillator on the flexural wave vibration band gaps, the dimensionless spring stiffness *k_sdim_* = *k_s_*/*k_s0_* is defined to achieve dimensionless parameter study, where *k_s_*/*k_s0_* is the ratio of the spring stiffness to the spring stiffness *k_s0_* = 5.0 × 10^7^ N/m in the calculation example. Then the dispersion relations with different dimensionless spring stiffness values are performed. During the calculation, the material parameters and geometrical parameters of the proposed infinite long periodic oscillator coupled damping beam remain unchanged. With the gradually increase of the dimensionless spring stiffness *k_sdim_* from 10^−4^ to 10^4^, the flexural wave vibration band gaps map of the infinite long periodic oscillator coupled damping beam as functions of the factor of dimensionless spring stiffness *k_sdim_* is obtained as shown in Figure 5.

The dimensionless spring stiffness *k_sdim_* has significant influence on the flexural wave vibration band gaps. With the increase of the dimensionless spring stiffness *k_sdim_*, the initial frequencies, terminal frequencies and bandwidths of the band gaps increase in a multistep manner. For the first band gap of the proposed infinite long periodic oscillator coupled damping beam, with the increase of the dimensionless spring stiffness *k_sdim_* from 10^−^^4^~10^1^, the initial frequency and terminal frequency gradually increase, while when the *k_sdim_* in the range of 10^1^~10^4^, the initial frequency reaches about 458 Hz and then no longer changes, and the terminal frequency reaches about 592 Hz and then no longer changes; Thus, with the increase of the dimensionless spring stiffness *k_sdim_* from 10^−^^4^~5, the bandwidth gradually increases to the maximum value of 159.6 Hz when the *k_sdim_* = 5, and then decreases to about 134 Hz and no longer changes when the *k_sdim_* in the range of 10^1^~10^4^. This can be explained that the formation mechanism of the first band gap is mainly determined by the local resonance of periodic spring-mass oscillator, with the increase of the dimensionless spring stiffness *k_sdim_*, the eigenfrequencies of the periodic spring-mass oscillator increase.

For the second band gap, with the increase of the dimensionless spring stiffness *k_sdim_* from 10^−^^4^~10^−1^, the initial frequency and terminal frequency remains unchanged at about 583 Hz and 600 Hz, respectively, while when the *k_sdim_* in the range of 10^−1^~10^2^, the initial frequency and the terminal frequency respectively gradually increase to 1530 Hz and 1894 Hz, and then remain unchanged; Thus, with the increase of the dimensionless spring stiffness *k_sdim_* from 10^−^^4^~10^−1^, the bandwidth remains unchanged at about 17 Hz, and then gradually increases to the maximum value of 426.8 Hz when the *k_sdim_* = 5, after that the bandwidth decreases to about 360 Hz and no longer changes. This phenomenon is due to that the second band gap is caused by the coupling effect between the flexural wave vibration of the homogeneous straight damping beam and the Bragg scattering of the periodic oscillator. With the increase of the dimensionless spring stiffness *k_sdim_*, the reaction force of the spring-mass oscillator acting on the beam enhances and rises the bending stiffness of the beam, resulting in the increase of the eigenfrequencies of the proposed infinite long periodic oscillator coupled damping beam.

#### 3.3.2. Effect of Spring-Mass Oscillator Mass on the Band Gaps

**Figure 6:**
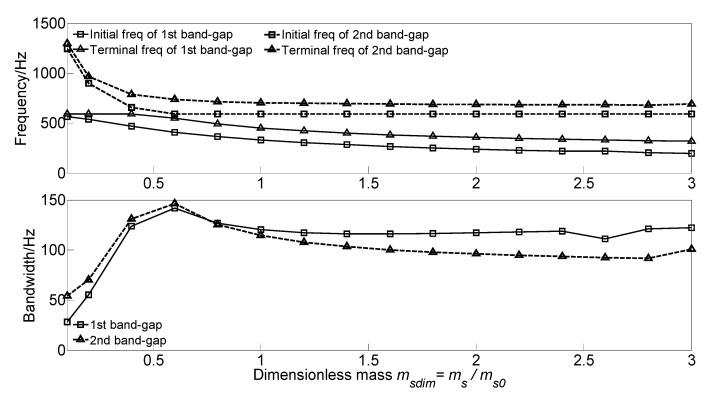
Band gaps maps as functions of dimensionless mass *m_sdim_*.

To analyze the effect of spring-mass oscillator mass *m_s_* on the flexural wave vibration band gaps of the proposed infinite long periodic oscillator coupled damping beam, we keep the material parameters and geometrical parameters as same as those in the calculation example. The dimensionless mass *m_sdim_* = *m_s_*/*m*_*s*0_ is defined to achieve dimensionless parameter study, with the increase of the dimensionless mass *m_sdim_* from 0.1 to 3.0, the effect of mass on the band gaps are conducted, and the flexural wave vibration band gaps maps as functions of the factor of dimensionless mass *m_sdim_* are illustrated in Figure 6.

It can be found that, with the increase of the dimensionless mass *m_sdim_* from 0.1 to 3.0, the initial frequency and terminal frequency of the two band gaps shift to a low-frequency region with different forms. For the first band gap of the proposed infinite long periodic oscillator coupled damping beam, the initial frequency decreases from 566.1 to 198.0 Hz, and the terminal frequency remains unchanged at about 592 Hz when the *m_sdim_* in the range 0.1~0.4, and then gradually decreases to 320.3 Hz with the increase of the dimensionless mass *m_sdim_* from 0.4 to 3.0. Thus, the bandwidth increases to the maximum value of 141.7 Hz when the *m_sdim_* = 0.6, and gradually decreases to 116.2 Hz when the *m_sdim_* in the range 0.6~1.4, and then remains unchanged at about 116 Hz. This can be explained that the formation mechanism of the first band gap is mainly determined by the local resonance of periodic spring-mass oscillator; with the increase of the dimensionless mass *m_sdim_*, the eigenfrequencies of the periodic spring-mass oscillator decrease.

For the second band gap, with the increase of the dimensionless mass *m_sdim_*, the initial frequency decreases from 1243.8 to 591.4 Hz and then remains unchanged at about 591 Hz, and like that, the terminal frequency decreases from 1297.9 to 705.7 Hz and then remains unchanged at about 690 Hz. Thus, the bandwidth increases to the maximum value of 141.7 Hz when the *m_sdim_* = 0.6, and then slowly decrease to 90 Hz. This phenomenon is because the second band gap is caused by the coupling effect between the flexural wave vibration of the homogeneous straight damping beam and the Bragg scattering of the periodic oscillator. With the increase of the dimensionless mass *m_sdim_*, the reaction force of the spring-mass oscillator acting on the beam recedes and reduces the beam’s bending stiffness, resulting in the decrease of the eigenfrequencies.

#### 3.3.3. Effect of Lattice Constant on the Band Gaps

**Figure 7:**
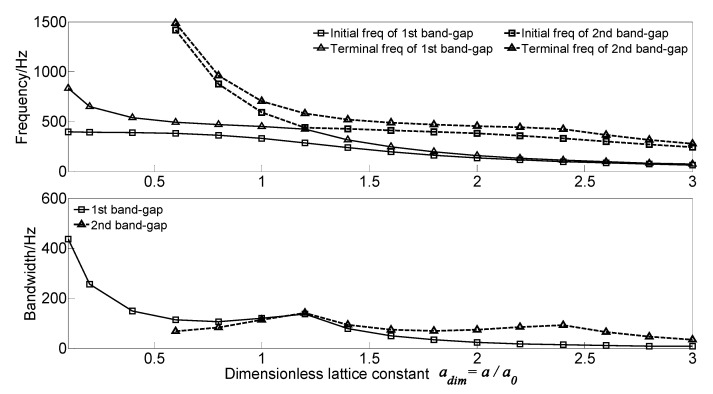
Band gaps maps as functions of dimensionless lattice constant *a_dim_*.

To analyze the effect of lattice constant on the flexural wave vibration band gaps of the proposed infinite long periodic oscillator coupled damping beam, the dimensionless lattice constant *a_dim_* = *a*/*a*_0_ is defined to achieve dimensionless parameter study, where *a*_0_ = 0.5 m is the lattice constant in the calculation example. With the increase of the dimensionless lattice constant *a_dim_* from 0.1 to 3.0, the flexural wave vibration band gaps map as functions of the dimensionless lattice constant *a_dim_* are calculated as shown in Figure 7.

As the dimensionless lattice constant *a_dim_* increases from 0.1 to 3.0, the two band gaps’ initial frequency and terminal frequency shift to the low-frequency region. For the first band gap of the proposed infinite long periodic oscillator coupled damping beam, the initial frequency decreases from 396.6 to 65.0 Hz, and the terminal frequency decreases from 832.9 to73.1 Hz. Thus, the bandwidth’s changing trend is decreasing, which decreases from 436.3 to 8.1 Hz and obtains a local maximum value of 136.3 Hz when the dimensionless lattice constant *a_dim_* = 1.2. It is clear that the second band gap’s changing trend is similar to that of first band gap, the initial frequency, and terminal frequency are much higher than 1500 Hz when the dimensionless lattice constant *a_dim_* in the range 0.1~0.6 and decreases to 243.8 and 279.0 Hz, respectively. The bandwidth of the second band gap increases first and gets the maximum value of 142.1 Hz. After that, the bandwidth gradually decreases to the local maximum value of 93.3 Hz when the dimensionless lattice constant *a_dim_* = 2.4. This can be explained that the band gaps’ formation mechanism is determined by the Bragg scattering of the periodic spring-mass oscillator. According to the Bragg scattering theory, the wavelengths corresponding to the intermediate frequencies of the band gaps in periodic structures are approximately twice the lattice constant.

#### 3.3.4. Effect of Spring-Mass Oscillator Damping on the Band Gaps

**Figure 8:**
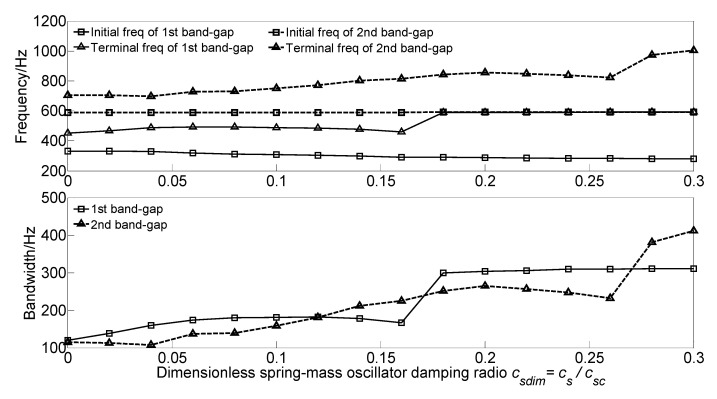
Band gaps maps as functions of dimensionless spring-mass oscillator damping radio *c_sdim_*.

To investigate the effect of spring-mass oscillator damping *c_s_* on the flexural wave vibration band gaps, the dimensionless spring-mass oscillator damping radio *c_sdim_* = *c_s_*/*c_sc_* is defined to achieve dimensionless parameter study, where $c_{sc}=2\sqrt{k_{s}m_{s}}=40,000\;N\cdot s/m$ is the critical damping of the spring-mass oscillator. With the increase of the dimensionless spring-mass oscillator damping radio *c_sdim_* from 0 to 0.3, the flexural wave vibration band gaps map as functions of the factor of dimensionless spring-mass oscillator damping radio *c_sdim_* are obtained as shown in Figure 8.

It can be found that, with the increase of the dimensionless spring-mass oscillator damping radio *c_sdim_* from 0 to 0.3, the initial frequencies of the two band gaps almost remain unchanged, whereas the terminal frequencies of the two band gaps increase continually and have a significant increase. Thus, the bandwidths gradually increase and significantly increase when the *c_sdim_* = 0.18 and *c_sdim_* = 0.28, respectively. This phenomenon is because the flexural wave vibration band gaps are caused by the coupling effect between the flexural wave vibration of the homogeneous straight damping beam and the Bragg scattering of the periodic oscillator. With the increase of the dimensionless spring-mass oscillator damping radio *c_sdim_*, the spring-mass oscillator’s reaction force acting on the beam enhances and rises the bending stiffness of the beam, increasing the eigenfrequencies.

#### 3.3.5. Effect of Beam Damping on the Band Gaps

**Figure 9:**
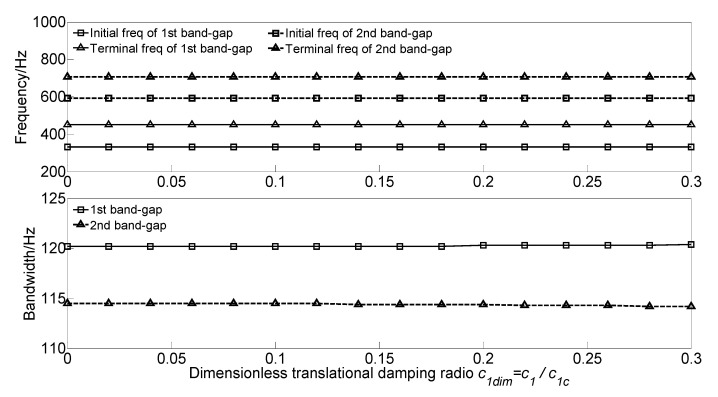
Band gaps maps as functions of dimensionless translational damping radio *c_1dim_*.

**Figure 10:**
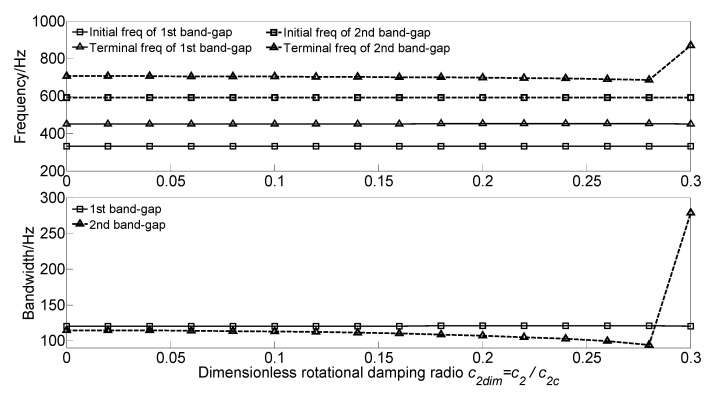
Band gaps maps as functions of dimensionless rotational damping radio *c_2dim_*.

To study the effect of translational damping *c_1_* and rotational damping *c*_2_ of the homogeneous straight damping beam on the flexural wave vibration band gaps, the dimensionless translational damping radio *c_1dim_* = *c*_1_/*c_1c_* and dimensionless rotational damping radio *c*_2*dim*_ = *c*_2_/*c_2c_* are defined to achieve dimensionless parameter study, where $c_{1c}=c_{2c}=2\pi^{2}\sqrt{EI\rho A}/L^{2}\approx 5000\;N\cdot s/m$ is the critical translational damping and critical rotational damping, respectively. With the increase of the dimensionless translational damping radio *c_1dim_* or the dimensionless rotational damping radio *c_2dim_* from 0 to 0.3, the flexural wave vibration band gaps maps as functions of the factor of dimensionless translational damping radio *c_1dim_* and dimensionless rotational damping radio *c_2dim_* are obtained as shown in Figure 9 and Figure 10, respectively.

It can be observed that, as the dimensionless translational damping radio *c_1dim_* increases, the initial frequencies and terminal frequencies of the two band gaps remain unchanged. Furthermore, the initial frequencies and terminal frequencies of the two band gaps almost remain unchanged when the dimensionless rotational damping radio *c_2dim_* increases from 0 to 0.3. This phenomenon is due to damping’s working principle; the damping absorbs and consumes the energy of the flexural wave vibration but did not change the frequency.

#### 3.3.6. Effect of Section Moment of Inertia on the Band Gaps

**Figure 11:**
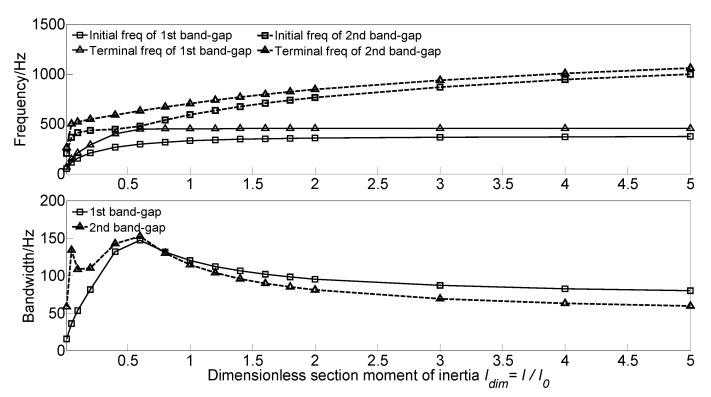
Band gaps maps as functions of dimensionless section moment of inertia *I_dim_*.

To investigate the effect of section moment of inertia *I* of the homogeneous straight damping beam on the flexural wave vibration band gaps of the proposed infinite long periodic oscillator coupled damping beam, the dimensionless section moment of inertia *I_dim_* = *I*/*I*_0_ is defined to achieve dimensionless parameter study. With the increase of the dimensionless section moment of inertia *I_dim_* from 0.01 to 5.0, the flexural wave vibration band gaps map as functions of the factor of dimensionless section moment of inertia *I_dim_* are obtained as shown in Figure 11.

It can be found that, with the increase of the dimensionless section moment of inertia *I_dim_* from 0.01 to 5.0, the initial frequencies and terminal frequencies of the two band gaps move up to the high-frequency region. For the first band gap, the initial frequency gradually increases from 50 to 374.9 Hz. the terminal frequency increase from 66.3 to 446.5 Hz first when the dimensionless section moment of inertia *I_dim_* in the range 0.01~0.6, and after that remains unchanged at about 450 Hz. Thus, the bandwidth increases from 15.5 Hz to the maximum value of 147.4 Hz when the dimensionless section moment of inertia *I_dim_* = 0.6, and then decreases to 80 Hz. For the second band gap, the initial frequency and terminal frequency significantly increase when the dimensionless section moment of inertia *I_dim_* in the range 0.01~0.1, which increases from 205.3 to 412.5 Hz and 263.9 to 520.9 Hz, respectively. Furthermore, after that, they gradually increase to 1000.5 and 1059.7 Hz. Thus, the bandwidth increases to 133.7 Hz first when the dimensionless section moment of inertia *I_dim_* = 0.05 and then decreases to 108.4; after that, the bandwidth increases to the maximum value of 152.4 Hz and gradually decreases to 59 Hz at last. This phenomenon is because the flexural wave vibration band gaps are caused by the coupling effect between the flexural wave vibration of the homogeneous straight damping beam and the Bragg scattering of the periodic oscillator. With the increase of the dimensionless section moment of inertia *I_dim_*, the homogeneous straight damping beam’s bending stiffness enhances, increasing the eigenfrequencies.

#### 3.3.7. Effect of Section Area on the Band Gaps

**Figure 12:**
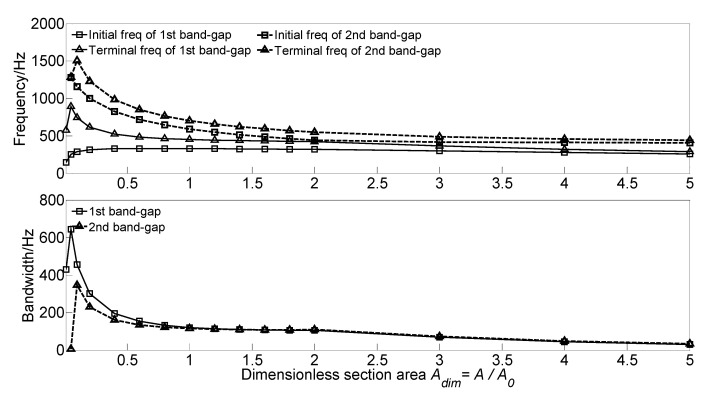
Band gaps maps as functions of dimensionless section area *A_dim_*.

To investigate the effect of section area *A* of the homogeneous straight damping beam on the flexural wave vibration band gaps, the dimensionless section area *A_dim_* = *A*/*A_0_* is defined to achieve dimensionless parameter study. With the increase of the dimensionless section area *A_dim_* from 0.01 to 5.0, the flexural wave vibration band gaps map as functions of dimensionless section area *A_dim_* are obtained as shown in Figure 12.

With the increase of the dimensionless section area *A_dim_* from 0.01 to 5.0, the initial frequency and terminal frequency of the first band gap move up to the high-frequency region, whereas those of the second band gap shift to the low-frequency region. For the first band gap, the initial frequency increased from 146.2 to 313.9 Hz when the dimensionless section area *A_dim_* in the range 0.01~0.2. It then gradually increases to the maximum value of 147.4 Hz when the dimensionless section area *A_dim_* = 0.6; after that, the initial frequency gradually decreases to 259.6 Hz. The terminal frequency also has a significant increase first from 575.3 Hz to the maximum value of 894.8 Hz, and then gradually decreases to 289.6 Hz. Thus, the bandwidth has a significant increase first from 429.1 Hz to the maximum value of 644.0 Hz and then significantly decreases to 120.2 Hz when the dimensionless section area *A_dim_* in the range 0.05~1.0, and gradually decreases to 30 Hz at last. For the second band gap of the infinite long periodic oscillator coupled damping beam, with the increase of dimensionless section area *A_dim_* from 0.01 to 5.0, the initial frequency gradually decreases from 1280.1 to 406.4 Hz when the dimensionless section area *A_dim_* increases to 5.0, and the terminal frequency increases from 1286.1 Hz to the maximum value of 1504.2 Hz, and then gradually decreases to 440.9 Hz. Thus, the bandwidth increases to the maximum value of 345.1 Hz, and gradually decreases to 34.5 Hz at last. This phenomenon is because the flexural wave vibration band gaps are caused by the coupling effect between the flexural wave vibration of the homogeneous straight damping beam and the Bragg scattering of the periodic oscillator. With the increase of the dimensionless section area *A_dim_*, the homogeneous straight damping beam’s bending stiffness reduces, resulting in a decrease of the eigenfrequencies.

#### 3.3.8. Effect of Material Parameters on the Band Gaps

**Figure 13:**
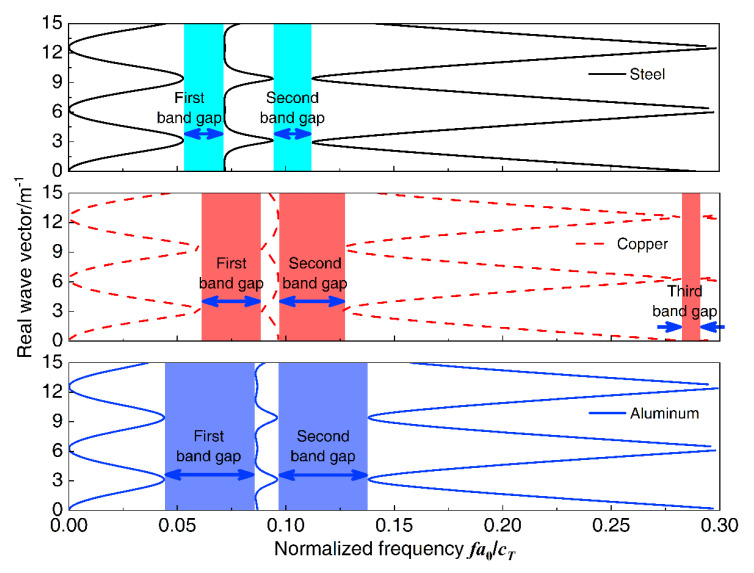
The band gaps of different homogeneous straight damping beam material properties.

**Figure 14:**
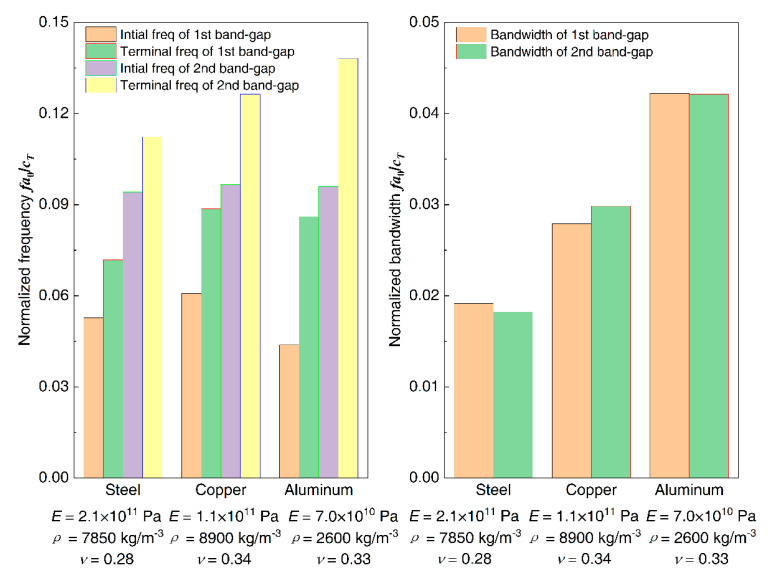
The influence of the material properties on band gaps.

To study the effect of material parameters of the proposed homogeneous straight damping beam on the flexural wave vibration band gaps, change the material of the beam as steel, copper, and aluminum, and then the dispersion relations were conducted. For the calculation, the material parameters of the proposed homogeneous straight damping beam are shown in Table 2, and the other geometrical parameters of the infinite long periodic oscillator coupled damping beam were the same as listed in Table 1. The flexural wave vibration band gaps of different homogeneous straight damping beam material properties as steel, copper, and aluminum are shown in Figure 13, respectively. The influence of material properties on band gaps is shown in Figure 14. The normalized frequency $fa_{0}/c_{T}$ is defined to achieve more versatile results, where *f* is the wave frequency and $c_{T}$ is the transversal wave speed of steel, copper, or aluminum, which the values are 3140, 2260, and 3080 m/s, respectively.

It can be found that, in the normalized frequency range from 0 to 0.3, it has two band gaps when the material of the beam is steel and aluminum, whereas there are three band gaps when the beam is copper. For the first band gap, the initial frequency increases when the material changes from steel to copper and shifts to a much lower-frequency region when the material is aluminum, the terminal frequencies are similar when the material is copper and aluminum, and higher than that when the material is steel. Thus, the bandwidths increase when the material changes from steel to copper and then changes to aluminum. For the second band gap, the initial frequencies are almost similar, and the terminal frequencies gradually increase; thus, the bandwidths gradually increase when the material changes from steel to copper and then changes to aluminum.

## 4. Conclusions

In this paper, the flexural wave vibration band gaps calculation method of the infinite long periodic oscillator coupled damping beam is derived by using the method of the reverberation-ray matrix in combination with the Bloch theorem, and the numerical results expressed by the frequency response function demonstrated the effectiveness of this theoretical calculation method. Parametric studies show that the flexural wave vibration band gaps can be artificially modulated and optimized by tuning the geometrical parameters and material parameters. The main conclusions are as follows:

1. As the study of the effect of spring stiffness on the band gaps, the initial frequencies, terminal frequencies, and bandwidths of the band gaps increase in a multistep manner. The band gaps’ frequencies have a significant increase when the spring stiffness in the range of 10^5^~10^8^ N·m^2,^ which is equivalent to the beam stiffness *EI* = 4.2 × 10^6^ N·m^2^, while the band gaps characteristics almost remain unchanged when the spring stiffness outside the range.
2. The dimensionless mass *m_sdim_* increase cause the frequencies of the band gaps gradually decrease, while there is a critical dimensionless mass value *m_sdim_* = 0.6, the terminal frequency of the first band gap remains unchanged when the dimensionless mass *m_sdim_* < 0.6, and the initial frequency and terminal frequency of the second band gap are almost unchanged when the dimensionless mass *m_sdim_* > 0.6.
3. As the damping absorbs and consumes the flexural wave vibration energy but did not change the spectrum characteristics, the translational damping *c*_1_ and rotational damping *c*_2_ of the homogeneous straight damping beam have little effect on the band gaps characteristics. While the effects of spring-mass oscillator damping on the band gaps are due to the proposed damping changes in the reaction force of the spring-mass oscillator acting on the beam.
4. The effects of section moment of inertia *I* and section area *A* on the band gaps are similar; the band gaps have significant changes when the dimensionless parametric ratios are less than the critical values, whereas almost remain unchanged when the dimensionless parametric ratios are greater than the critical values. Thus, it is not conducive to applying band gap characteristics to the structural vibration reduction design in engineering.
5. The spring stiffness *k_s_* and lattice constant *a* have great effects on band gaps characteristics; with the increase of the dimensionless spring stiffness and dimensionless lattice constant, the initial frequencies and terminal frequencies gradually change. This is conducive to applying band gap characteristics by tuning the geometrical parameters for structural vibration reduction design.

As a preliminary study, this paper provides an efficient theoretical approach to study the band gaps characteristics of 1D periodic structures. The detailed parametric studies are of great guiding significance for the vibration reduction design of periodic beam structures and the equivalent 2D periodic stiffened plate structures, such as the 1D periodic structures in high-speed railway, building foundations, bridge structures, ships and offshore structures, and the 2D periodic stiffened plates in bridge structures, ships, and offshore structures. Further investigations will address the proposed theoretical approach to study the vibration reduction design of these periodic structures or extend the proposed method to investigate the effects of nonperiodic factors on band gaps characteristics; the nonperiodic factors would include the concentrated loads, lumped mass, structural changes, and even the influence of manufacturing precision in engineering.

## Figures and Tables

**Table 1:** The parameters of homogeneous straight damping beam and spring-mass oscillator in the calculation example, the subscript 0 is defined to distinguish the calculation example and parametric study.

Parameter	*k*_*s*0_	*m*_*s*0_	*a*_0_	*c*_*s*0_	*c*_1_	*c*_2_	*I*_0_	*A*_0_
Value	5.0 × 10^7^ N/m	8 kg	0.5 m	0 N·s/m	0 N·s/m	0 N·s/m	2.0 × 10^−5^ m^4^	3.0 × 10^−3^ m^2^

**Table 2:** The parameters of the beam and spring-mass oscillator.

	Steel	Copper	Aluminum
Young’s modulus E/Pa	2.1 × 10^11^	1.1 × 10^11^	7.0 × 10^10^
Mass density *ρ*/kg·m^−3^	7850	8900	2600
Poisson’s ratio *ν*	0.28	0.34	0.33

Note: Other geometrical parameters were listed in Table 1.
